# Anti-allodynic and promotive effect on inhibitory synaptic transmission of riluzole in rat spinal dorsal horn

**DOI:** 10.1016/j.bbrep.2021.101130

**Published:** 2021-09-10

**Authors:** Ryo Taiji, Manabu Yamanaka, Wataru Taniguchi, Naoko Nishio, Shunji Tsutsui, Terumasa Nakatsuka, Hiroshi Yamada

**Affiliations:** a Department of Orthopaedic Surgery, Wakayama Medical University, 811-1 Kimiidera, Wakayama, 641-8510, Japan; b Pain Research Center, Kansai University of Health Sciences, 2-11-1 Wakaba, Kumatori, Osaka, 590-0482, Japan

**Keywords:** Spinal dorsal horn, Nociceptive transmission, Riluzole, Mechanical allodynia, Neuropathic pain, CCI, chronic constriction injury, SG, substantia gelatinosa, SNI, spared nerve injury, TTX, tetrodotoxin, IPSC, inhibitory postsynaptic current

## Abstract

Riluzole (2-amino-6-(trifluoromethoxy)benzothiazole) is a drug known for its inhibitory effect on glutamatergic transmission and its anti-nociceptive and anti-allodynic effects in neuropathic pain rat models. Riluzole also has an enhancing effect on GABAergic synaptic transmission. However, the effect on the spinal dorsal horn, which plays an important role in modulating nociceptive transmission, remains unknown. We investigated the ameliorating effect of riluzole on mechanical allodynia using the von Frey test in a rat model of neuropathic pain and analyzed the synaptic action of riluzole on inhibitory synaptic transmission in substantia gelatinosa (SG) neurons using whole-cell patch clamp recordings. We found that single-dose intraperitoneal riluzole (4 mg/kg) administration effectively attenuated mechanical allodynia in the short term in a rat model of neuropathic pain. Moreover, 300 μM riluzole induced an outward current in rat SG neurons. The outward current induced by riluzole was not suppressed in the presence of tetrodotoxin. Furthermore, we found that the outward current was suppressed by simultaneous bicuculline and strychnine application, but not by strychnine alone. Altogether, these results suggest that riluzole enhances inhibitory synaptic transmission monosynaptically by potentiating GABAergic synaptic transmission in the rat spinal dorsal horn.

## Highlights

• Riluzole shows anti-nociceptive and anti-allodynic effects in neuropathic pain models.
• The spinal dorsal horn plays an important role in modulating nociceptive transmission.
• Riluzole potentiated GABAergic synaptic transmission in the rat spinal dorsal horn.
•Riluzole enhanced inhibitory synaptic transmission monosynaptically.

## 1 Introduction

Persistent neuropathic pain conditions, including allodynia, hyperalgesia, and spontaneous pain, which result from injury or inflammation of the peripheral nerves, significantly reduce the ability to perform activities of daily living and quality of life [1,2]. While analgesics acting through a range of mechanisms have been developed, there are still many patients for whom sufficient analgesic effects have not been achieved with existing drugs. Therefore, the development of new therapeutic agents for neuropathic pain is needed.

Riluzole (2-amino-6-(trifluoromethoxy)benzothiazole), a U.S. Food and Drug Administration-approved drug for the treatment of amyotrophic lateral sclerosis [3], has been demonstrated to attenuate neural excitotoxicity by inhibiting glutamatergic transmission. This involves multiple mechanisms, including attenuation of presynaptic glutamate release via blockade of both sodium and calcium channels [[4], [5], [6]], enhancement of glutamate transporter activity [[7], [8], [9]] and weak antagonism of post-synaptic NMDA receptors [4,10]. This drug has also been reported to have anti-nociceptive and anti-allodynic effects in rat models of chronic constriction injury (CCI) and in other pain models [8,[11], [12], [13], [14]]. It has been reported that 1–4 mg/kg of riluzole reduced heat hyperalgesia and mechanical allodynia in CCI rats when administered intrathecally, twice daily, either during days 1–4 or during days 5–8 [8]. In addition, 6 and 12 mg/kg of riluzole produced a prolonged attenuation of both mechanical and cold hypersensitivity in CCI rats when administered intraperitoneally, twice daily, during days 1–4 [11]. These pain relief mechanisms are supported by previous evidence showing that pain due to nerve injury depends on glutamate release and cellular changes associated with excessive glutamate neurotransmission [15,16]. On the other hand, it is also known that riluzole promotes GABAergic synaptic transmission [17,18], which might contribute to the anti-nociceptive and anti-allodynic properties of this pharmacological agent. However, the mechanisms underlying the effects on spinal inhibitory synaptic transmission remain unclear. In particular, the effects on GABAergic neurotransmission in the spinal dorsal horn have not been investigated yet.

In the present study, we employed integrative methods, including whole-cell patch clamp recording, pharmacology, and behavioral tests, to investigate the ameliorating effect of riluzole in a neuropathic pain model, and analyzed the synaptic action of riluzole on inhibitory synaptic transmission in the spinal dorsal horn. We showed that single-dose riluzole (4 mg/kg) effectively attenuated mechanical allodynia in short term in SNI model, and that 300 μM of riluzole induced an outward current in rat SG neurons. Furthermore, we found that riluzole enhances inhibitory synaptic transmission monosynaptically by potentiating GABAergic synaptic transmission in rat spinal dorsal horn.

## 2 Materials and methods

### 2.1 Animals

Five to six-week-old male Sprague Dawley rats (140–260 g; obtained from Kiwa Laboratory Animals Co., Japan) were used. The protocol was approved by the Ethics Committee on Animal Experiments of Wakayama Medical University and conducted in accordance with the U.K. Animals (Scientific Procedures) Act of 1986 and associated guidelines. Animals were housed in plastic cages at room temperature on a 12-h light/dark cycle (light on between 8:00 a.m. and 8:00 p.m.) with ad libitum access to food and water.

### 2.2 Neuropathic pain model

Under isoflurane anesthesia (2–3%), the skin on the lateral surface of the thigh was incised, and the sciatic nerve and its three terminal branches (sural, common peroneal, and tibial nerves) exposed. The spared nerve injury (SNI) procedure consisting in an axotomy and ligation of the tibial and common peroneal nerves leaving the sural nerve intact was performed [19]. The common peroneal and tibial nerves were tightly ligated with 4-0 silk sutures and sectioned distal to the ligation. Great care was taken to avoid any contact with or stretching of the intact sural nerve. The muscle and skin were closed in two layers.

### 2.3 Drug preparation and administration

Riluzole (Wako, Osaka, Japan) was dissolved in 5% dimethyl sulfoxide (DMSO), resulting in a concentration of 4 mg/kg, diluted to a final volume of 1 ml. All injured rats were divided blindly and randomly into two experimental groups: (1) riluzole group (riluzole injection, n = 10), and (2) control group (DMSO in saline injection, n = 10). In the riluzole group, riluzole was intraperitoneally administered once at 7 days after surgery. In the control group, the same amount of DMSO in saline was intraperitoneally administered on the same day as with the riluzole group.

### 2.4 Behavioral test for evaluation of neuropathic pain

The experimenter was blinded to drug status. The hind paw withdrawal threshold was measured as the frequency of foot withdrawals elicited by a defined mechanical stimulus using a 10 g von Frey filament [20,21]. Animals were habituated to the environment and behavioral tests for three days prior to the surgery described above. The rats were placed in a chamber measuring 18 × 25 × 18 cm above the wire mesh floor. They were acclimatized to the environment and investigator for ≥20 min before the test. The mechanical stimulus was applied to the lateral area of the plantar surface (area perceived by the sural nerve) of the ipsilateral hind paw and maintained for approximately 2 s. A withdrawal response was considered valid only if the hind paw was completely removed from the platform. The hind paw was probed consecutively with 10 stimulations, and the frequency of withdrawal responses evaluated. The evaluations were performed at 1, 3, 5, and 7 days after surgery. Seven days after surgery, the evaluation tests were performed before drug administration and at 1, 6, 12, and 24 h after administration.

### 2.5 In vitro whole-cell patch-clamp recording

We used naïve rats for patch-clamp recordings. The methods used to obtain the transverse spinal cord slices have been described previously [22]. Briefly, the rats were anesthetized with urethane (1.2–1.5 g/kg, intraperitoneal), and Th10-L4 laminectomy was performed. We then excised the lumbosacral spinal cord and submerged it in preoxygenated Krebs' solution at 1–3 °C. Immediately after the removal of the spinal cord, the rats were killed by exsanguination under urethane. The dura, arachnoid, and pia mater were removed, and the spinal segment was sliced with a 650-μm thickness by using a microslicer (DTK-1000, Dousaka, Kyoto, Japan). The slices were continuously perfused with Krebs' solution (5–10 ml/min) and saturated with 95% O_2_ and 5% CO_2_. The perfusion solution was heated to 36 ± 1 °C using a temperature controller. The Krebs' solution contained 117 mM NaCl, 3.6 mM KCl, 1.2 mM NaH_2_PO_4_, 2.5 mM CaCl_2_, 1.2 mM MgCl_2_, 25 mM NaHCO_3_, and 11 mM glucose at pH 7.4.

Blind whole-cell patch-clamp recordings were prepared from lamina II substantia gelatinosa (SG) neurons in the voltage-clamp mode, as described previously [[23], [24], [25]]. Experiments were performed in a recording chamber with a microscope for visualization of the whole-cell patch-clamp recording. The patch pipette had a resistance of 5–10 Ω, and the composition of the patch-pipette internal solution was as follows: 110 mM Cs_2_SO_4_, 5 mM tetraethylammonium, 0.5 mM CaCl_2_, 2 mM MgCl_2_, 5 mM EGTA, 5 mM ATP-Mg, 5 mM HEPES-KOH; pH 7.2, 305 mOsm. The holding potential was set to 0 mV to record the IPSCs. Signals were collected using an Axopatch 200 B amplifier in conjunction with a Digidata 1440A A/D converter (Molecular Devices, Sunnyvale, CA, USA) and stored on a personal computer using the pCLAMP 10 data acquisition program (Molecular Devices). Recordings were analyzed using Mini Analysis software (version 6.0; Synaptosoft, Fort Lee, NJ, USA) and pCLAMP 10. Membrane potential recordings were performed in the current-clamp mode.

### 2.6 Drug application

The drugs used in this study were riluzole (Wako, Osaka, Japan), tetrodotoxin (TTX) (Wako, Osaka, Japan), bicuculline (Sigma-Aldrich Co., St. Louis, USA), and strychnine (Sigma-Aldrich Co., St. Louis, USA). Riluzole, bicuculline, and strychnine were first dissolved in DMSO at 1000 × the final concentration, and TTX first dissolved in distilled water at 1000 × the final concentration. The drugs were then diluted to final concentrations in Krebs solution immediately before use and applied by perfusion via a three-way stopcock without any change in perfusion rate or temperature.

### 2.7 Statistical analysis

All numerical data were expressed as the mean ± standard error of the mean (SEM). Mechanical allodynia was analyzed using two-way ANOVA with time and drug administration as factors followed by Holm Sidak post hoc test. Student's t-test and Mann-Whitney *U* test were used to determine the statistical significance between means. *p* < 0.05 was considered significant for these tests. For electrophysiological data, n refers to the number of neurons studied.

## 3 Results

### 3.1 Anti-allodynic effect of riluzole on neuropathic pain in SNI rats

**Fig. 1:**
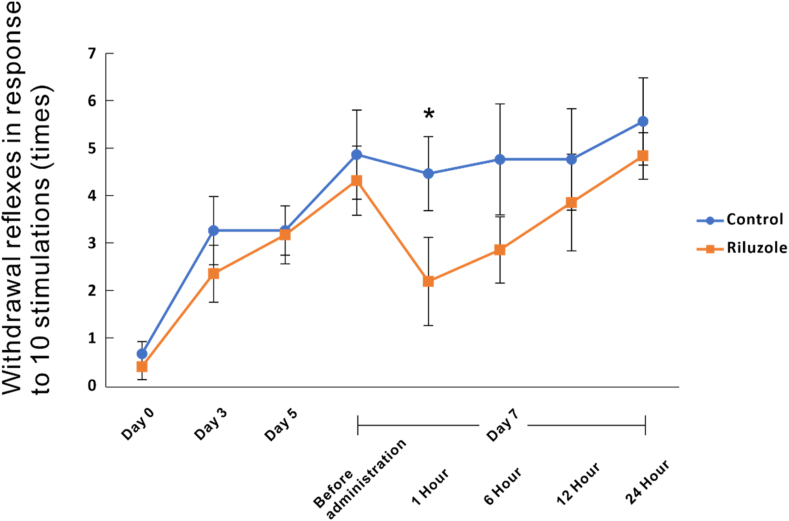
Effect of riluzole on mechanical allodynia in spared nerve injury (SNI) rats. The graph illustrates the changes in the number of withdrawal reflexes to mechanical stimulation using a 10 g von Frey filament following administration of riluzole and vehicle under mechanical allodynia by SNI. Riluzole group (n = 10) demonstrated significantly decreased withdrawal reflexes compared to the control group (n = 10) at 1 h after drug administration (*p < 0.05).

The number of withdrawal reflexes in the riluzole group (2.0 ± 0.9 times) significantly decreased compared with that in the control group (4.5 ± 0.8 times) (*p* < 0.05) at 1 h after drug administration (Fig. 1). However, the difference between the groups disappeared gradually, and there was no significant difference at 6 h or later after drug administration. The results of this experiment showed that a single dose of riluzole attenuated mechanical allodynia in the short term in a rat model of SNI.

### 3.2 Outward current induced by riluzole in SG neurons

**Fig. 2:**
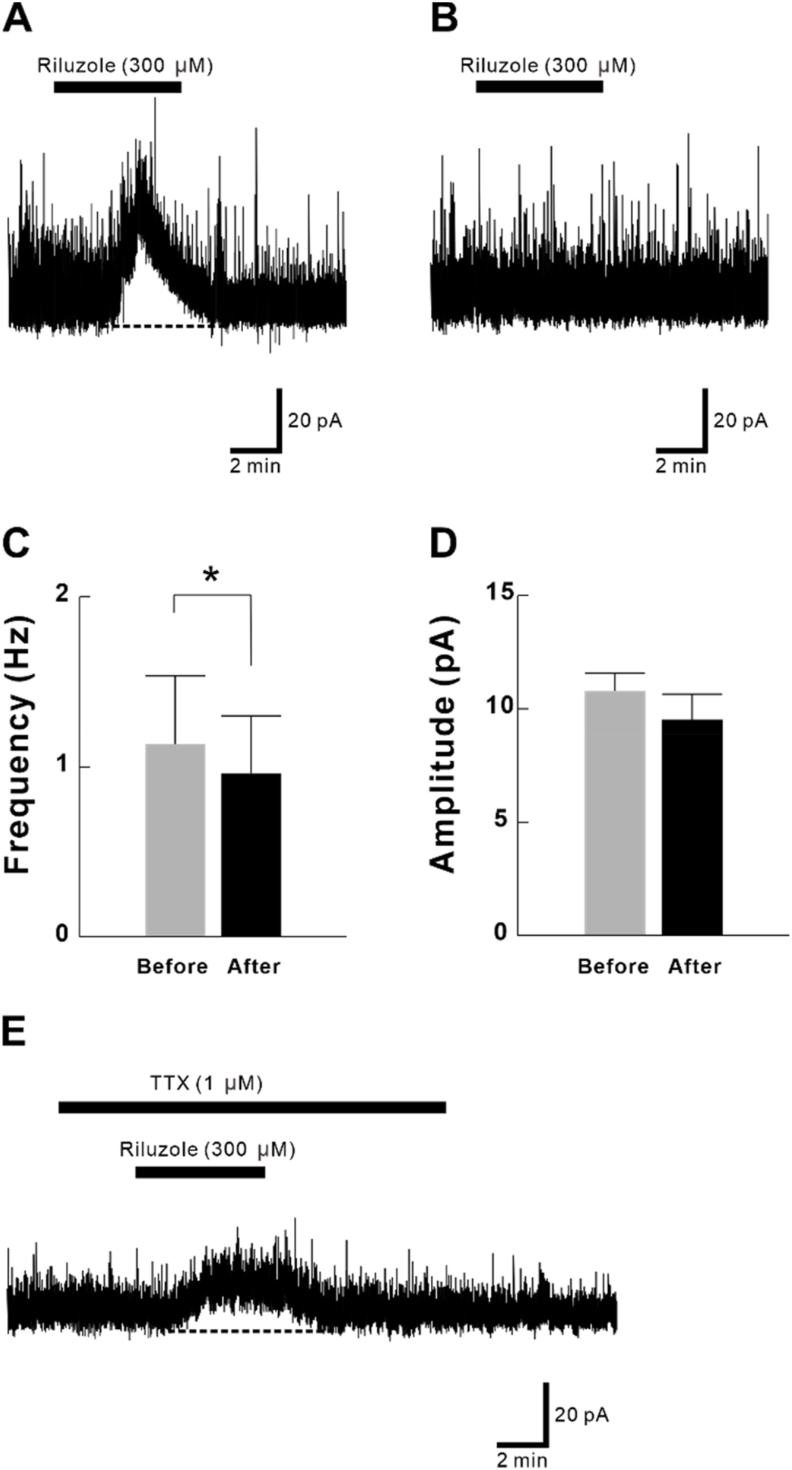
Outward IPSC currents induced by riluzole in substantia gelatinosa (SG) neurons. (A) Riluzole (300 μM) induced outward current (>5 pA) in some neurons (n = 8). (B) Riluzole (300 μM) did not induce outward current in the other neurons (n = 5). (C) IPSC frequency decreased by riluzole application (*p < 0.05). (D) IPSC amplitude did not change by riluzole application. (E) Outward current induced by riluzole in the presence of tetrodotoxin (TTX) (1 μM). TTX did not block the outward current (n = 4).

All neurons studied had membrane potentials <−50 mV, and each neuron was recorded from a different slice. Superfusion of riluzole (300 μM) for 5 min induced an outward current (>5 pA) in eight of the 13 SG neurons, and the mean peak outward current was 20.3 ± 3.9 (n = 8). Typical charts are shown in Fig. 2A and B. IPSC analysis showed a frequency and amplitude of IPSC of 1.14 ± 0.40 Hz and 10.8 ± 0.8 pA, respectively, before riluzole application, and 0.96 ± 0.34 Hz and 9.5 ± 1.1 pA, respectively, after riluzole application (Fig. 2C and D). IPSC frequency decreased significantly by riluzole administration. On the other hand, IPSC's rise time and decay time were 4.3 ± 0.7 msec and 16.8 ± 2.3 ms, respectively, before riluzole application, and 5.1 ± 1.3 msec and 17.5 ± 4.6 ms, respectively, after application. There are no significant differences between them. Although this outward current can be obtained even at 100 μM (in three out of the six neurons), the mean peak outward current was relatively small (10.0 ± 7.4 pA), hence we used 300 μM for later experiment in order to observe the reaction more clearly.

The outward current induced by riluzole was also observed in the presence of TTX (1 μM). The outward current was 17.7 ± 2.8 pA in the presence of TTX (n = 4), there was no significant difference compared to administration of riluzole alone. A typical chart is shown in Fig. 2E. It is suggested that riluzole causes outward current through a monosynaptic response.

**Fig. 3:**
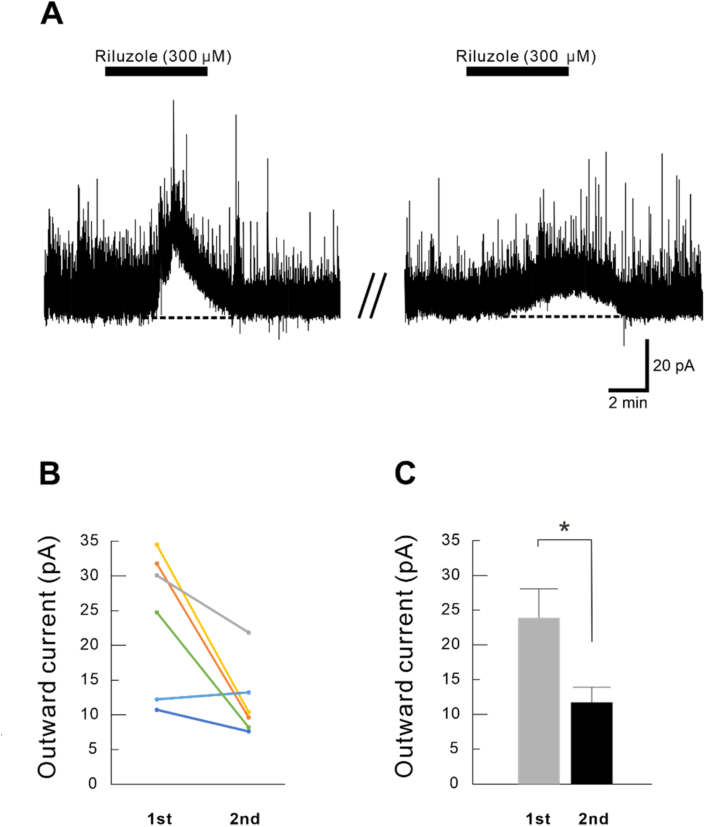
Reproducibility of the outward currents introduced by riluzole in substantia gelatinosa (SG) neurons. (A) The outward current induced by riluzole (300 μM) was reproducible when riluzole was applied twice in the same neuron (n = 6). (B) Reproducibility of the outward currents induced by riluzole for individual neurons. (C) Summary of outward current amplitude induced by the first and second riluzole administration (*p < 0.05).

Next, we investigated whether the outward current was reproducible when riluzole was applied again after 25 min of washout. The outward current induced by the second application (11.8 ± 2.2 pA) was decreased (*p* < 0.05) when compared with that of the first (24.0 ± 4.2 pA) (n = 6) (Fig. 3). This result indicated that riluzole could induce desensitization of its receptor.

### 3.3 Impact of GABAergic and glycinergic system on outward current of IPSC

**Fig. 4:**
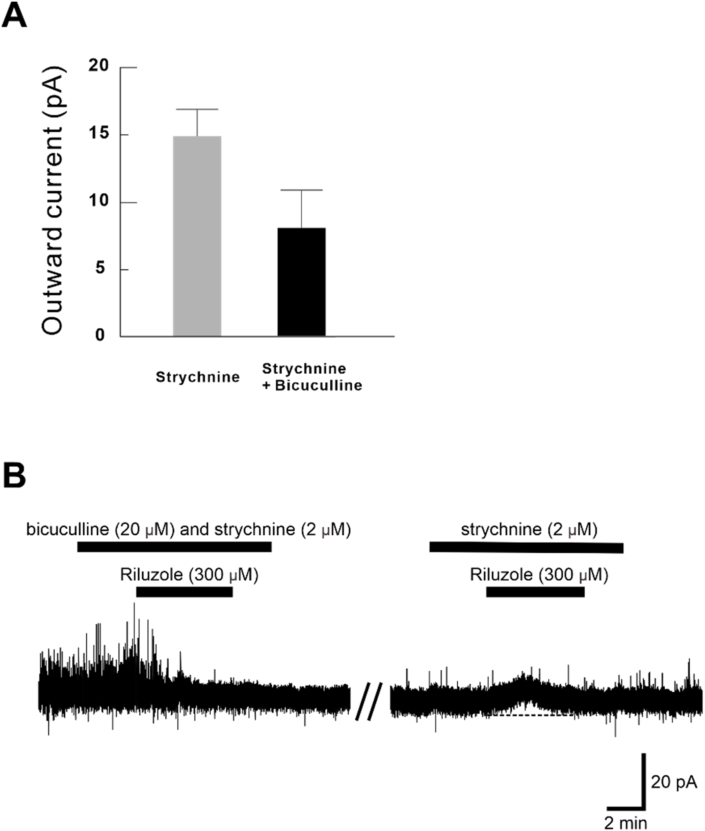
Outward current induced by riluzole in the presence of bicuculline and strychnine. (A) The amplitude of the outward current was lower in the presence of both bicuculline and strychnine (n = 2, two of the six neurons) than with strychnine alone (n = 4, four of the five neurons). (B) Although combined administration of bicuculline (20 μM) and strychnine (2 μM) blocked the outward current introduced by riluzole (300 μM), single administration of strychnine did not block the outward current in the same neuron.

Superfusion of riluzole induced an outward current in four of the five neurons (14.9 ± 2.0 pA) in the presence of the selective glycine receptor antagonist strychnine (2 μM). On the other hand, the outward current was obtained in only two of the six neurons (8.0 ± 2.8 pA) when riluzole is administrated with strychnine and the selective GABA_A_ receptor antagonist bicuculline (20 μM) (Fig. 4A). It also shows that although bicuculline and strychnine suppressed riluzole induced outward current, it was not suppressed when only strychinine was applied to the same neuron after 25 min of washout (Fig. 4B). These results indicated that the outward current induced by riluzole was caused by the GABAergic system.

## 4 Discussion

In the present study, we found that single-dose intraperitoneal riluzole administration effectively attenuated mechanical allodynia in the short term in SNI rats. The results of patch-clamp recording from SG neurons revealed that riluzole induced outward current, which was suppressed by bicuculline and strychnine when applied simultaneously, but not by strychnine alone. These results suggest that riluzole enhances inhibitory synaptic transmission by potentiating GABAergic synaptic transmission in the spinal dorsal horn.

Increasing evidence has confirmed the anti-nociceptive and anti-allodynic efficacies of riluzole in a rat model of neuropathic pain induced by chronic constriction injury [8,11,13]. It has been reported that riluzole (1–4 mg/kg) reduces heat hyperalgesia and mechanical allodynia in CCI rats when administered twice daily during days 1–4 or days 5–8. In this study, effects of early treatment lasted beyond treatment termination, with effective alleviation of hyperalgesia and allodynia extending to ≥10 days [8]. It has also been reported that riluzole (6 and 12 mg/kg) administered twice daily during days 1–4 in CCI rats reduces mechanical and cold allodynia until day 12 [11]. These results suggest not only that riluzole alleviates hyperalgesia and allodynia, but also that early treatment can prevent the development of heat hyperalgesia and mechanical allodynia. However, these studies reported relatively early intervention after nerve injury, while in the present study we evaluated the analgesic effect of mechanical allodynia in its complete development state by administering 4 mg/kg of riluzole at 7 days after surgery [19]. As a result, an attenuating effect was observed at 1 h after administration, but disappeared at 6 h after administration. This result is consistent with a previous report that the initial elimination half-life of a single dose of riluzole is 1.1–1.8 h [26]. It has also been reported that riluzole has a dose-response effect on thermal hyperalgesia [8]. Hence, one of the reasons for the short duration of anti-allodynic efficacy in this study could be the relatively low dose of administration.

We further investigated the synaptic response of riluzole in rat SG neurons, which plays an important role in modulating nociceptive transmission [27]. Using whole-cell patch-clamp recording, we showed that riluzole produced an outward current in rat SG neurons. Other studies have shown that the effect of riluzole on inhibitory neurotransmission by the GABAergic synaptic system. It has been shown that riluzole preferentially reduces glutamate release but also affects the transmission of other neurotransmitters, including GABA, by blocking presynaptic sodium channels [28,29]. Besides this presynaptic effect, riluzole has been shown to enhance GABA_A_ receptor function [18,[30], [31], [32]]. It has been reported that GABA_A_ currents evoked by application of lower concentrations of GABA (2 μM) to hippocampal neuron cultures or Xenopus oocytes expressing heteromeric GABA_A_ receptors α_1_β_2_γ_2_ are potentiated by lower concentrations of riluzole (20–300 μM) [18]. Miniature IPSC peak amplitude, rise time, and frequency were unaffected, indicating a postsynaptic mechanism. In addition, riluzole selectively increases the decay time constants of sIPSCs and has no effect on the peak amplitude, frequency, or rise time of the sIPSC in the DG region of the rat brain, indicating that the effect of riluzole on GABAergic inhibition is a postsynaptic mechanism [32]. In addition, riluzole inhibits GABA uptake in a dose-dependent manner and promotes synaptic GABA accumulation in rat striatal synaptosomes [17]. These reports indicate that riluzole potentiates GABAergic neurotransmission through several mechanisms in the central nervous system, but there are no reports focusing on its effect in the spinal cord. In the present study, we first revealed that riluzole affected GABAergic synaptic transmission in the spinal dorsal horn. Riluzole 300 μM produced an outward current in SG neurons, indicating enhancement of inhibitory neurotransmission. IPSC analysis showed that the IPSC frequency decreased with riluzole application, therefore, riluzole reduces GABA release as a presynaptic effect, while inducing an outward current in SG neurons. Hence, this outward current might be due to riluzole's postsynaptic effects. We also demonstrated that the outward current induced by riluzole was not a secondary effect of inhibiting glutamatergic transmission, as it was not suppressed by TTX. Furthermore, this outward current was suppressed by bicuculline and strychnine, but not by strychnine alone, indicating that it was a response to GABAergic neurotransmission. Hence, we suggest that riluzole also enhances the GABAergic system in the spinal dorsal horn. Although this outward current would be consistent with an accompanying membrane hyperpolarization, we cannot confirm this point because current-clamp recording was not conducted in this study, hence, it is the limitation of this study.

In conclusion, riluzole effectively attenuated mechanical allodynia in a rat SNI model. Mechanistically, riluzole enhances inhibitory synaptic transmission by inducing outward current by potentiating GABAergic synaptic transmission in the rat spinal dorsal horn.

## CRediT authorship contribution statement

**Ryo Taiji:** Writing – original draft, Formal analysis, Investigation, Visualization. **Manabu Yamanaka:** Writing – review & editing, Methodology, Supervision, Project administration. **Wataru Taniguchi:** Conceptualization, Methodology. **Naoko Nishio:** Investigation, Resources, Visualization. **Shunji Tsutsui:** Conceptualization. **Terumasa Nakatsuka:** Methodology, Software. **Hiroshi Yamada:** Supervision.

## Declaration of interests

The authors declare that they have no known competing financial interests or personal relationships that could have appeared to influence the work reported in this paper.
